# Corrigendum: A microfluidic paper-based electrochemical biosensor array for multiplexed detection of metabolic biomarkers (2013 *Sci. Technol. Adv. Mater.* 14 054402)

**DOI:** 10.1088/1468-6996/16/4/049501

**Published:** 2015-08-13

**Authors:** Chen Zhao, Martin M Thuo, Xinyu Liu

**Affiliations:** 1 Department of Mechanical Engineering, McGill University, 817 Sherbrooke Street West, MD270, Montreal, Quebec, H3A 0C3, Canada; 2 Department of Chemistry, University of Massachusetts—Boston, 100 Morrissey Blvd, Boston, MA 02125, USA

Figures 3, 4 and 5 appear incorrectly in this paper. Please see below the corrected versions of these figures.

**Figure 3.**
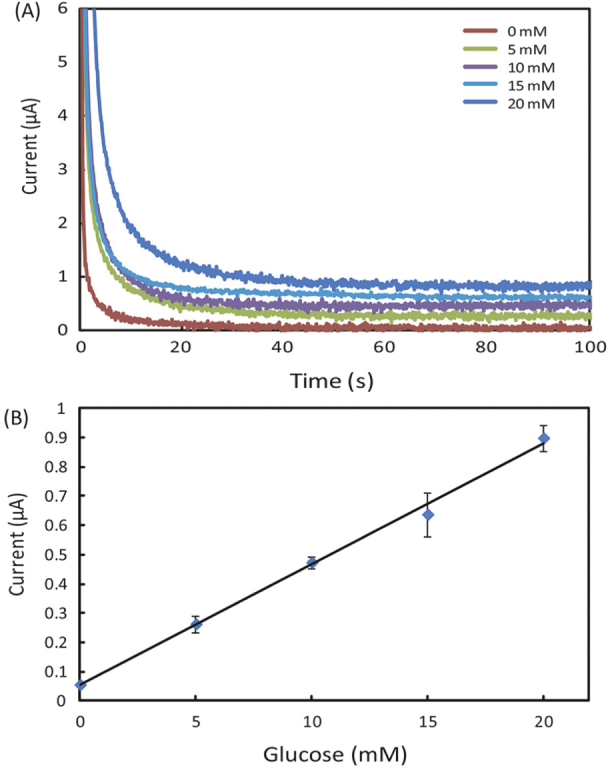
(A) Chronoamperometric curves and (B) the calibration plot for measurement of glucose in AU. The solid line in (B) represents a linear fit to experimental data with regression equation: *y* = 0.041*x* + 0.054 (*R*^2^ = 0.996, *n* = 5).

**Figure 4.**
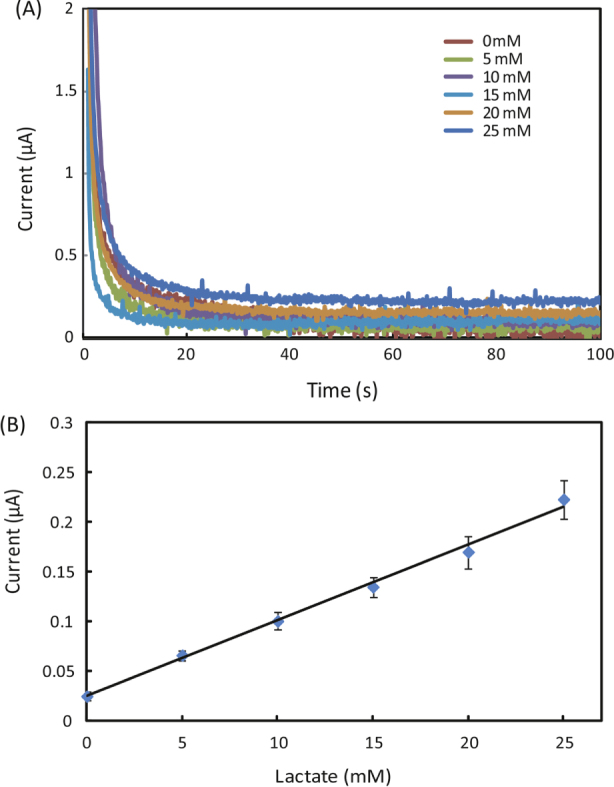
(A) Chronoamperometric curves and (B) the calibration plot for measurement of lactate in AU. The solid line in (B) represents a linear fit to experimental data with regression equation: *y* = 0.0076*x* + 0.025 (*R*^2^ = 0.995, *n* = 5).

**Figure 5.**
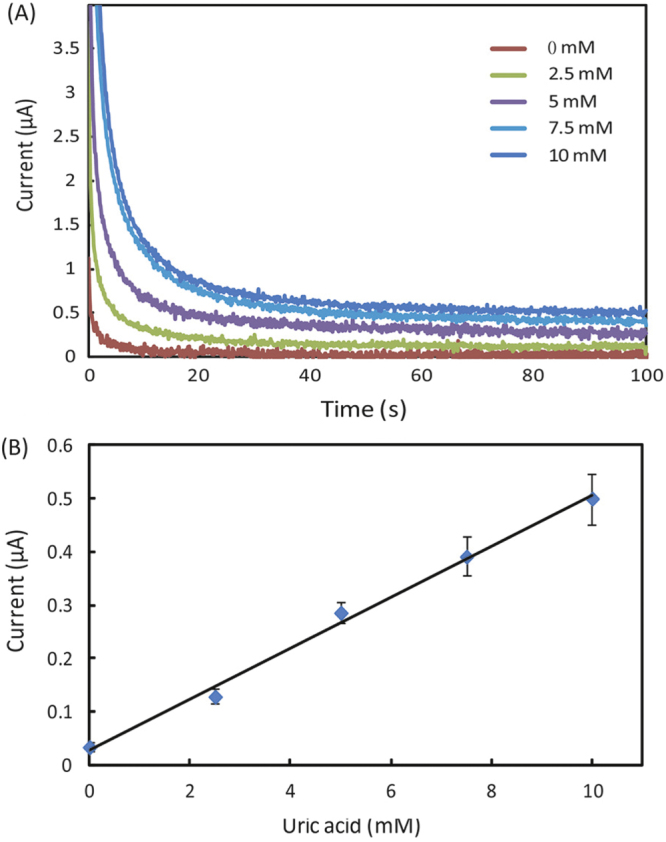
(A) Chronoamperometric curves and (B) the calibration plot for measurement of uric acid in AU. The solid line represents a linear fit to experimental data with regression equation: *y* = 0.048*x* + 0.029 (*R*^2^ = 0.994, *n* = 5).

